# Trend towards multiple authorship in occupational medicine journals

**DOI:** 10.1186/1745-6673-4-3

**Published:** 2009-02-09

**Authors:** Sami Shaban, Tar-Ching Aw

**Affiliations:** 1 Department of Community Medicine, Faculty of Medicine & Health Sciences, United Arab Emirates University, Al-Ain, UAE

## Abstract

**Background:**

There is an established trend towards an increasing number of authors per article in prestigious journals for medicine and health sciences. It is uncertain whether a similar trend occurs to the same extent in journals for specific medical specialties.

**Methods:**

Journals focusing on occupational medicine were selected for analysis with regard to single or multiple-authorship per peer-reviewed paper. Data were collected from PubMed for publications between 1970 and 2007. These were analysed to calculate the average number of authors per multiple-author article per year and the percentage of single-author articles per year. The slope and average of these journals were then compared with that of previously studied non-occupational medicine journals.

**Results:**

The results confirm a trend towards a linear increase in the average number of authors per article and a linear decrease in the percentage of single-author articles. The slope for the average number of authors for multiple-author articles was significantly higher in the Journal of Occupational and Environmental Medicine than in the other occupational medicine journals. Computational analysis of all articles published showed that Occupational Medicine (Oxford) had a significantly higher percentage of single-author articles than the other occupational medicine journals as well as major journals previously studied.

**Conclusion:**

The same trend towards multiple authorship can be observed in medical specialty journals as in major journals for medicine and health sciences. There is a direct relationship between occupational journals with higher impact factors and a higher average number of authors per article in those journals.

## Background

In academic circles, the publication of papers in peer-reviewed journals of high impact factor is highly regarded. The impact factor measures the frequency with which the average article in a specific journal is cited in a particular year. There are concerns regarding the relevance of the impact factor as a measure of quality [1], whether it unfairly disadvantages minor specialties in medicine and science [2], and whether it benefits journals more than authors or readers [3,4]. Despite these reservations, the focus on publications in high impact factor journals continues in many academic and research organizations. Alongside this has been a trend towards increasing the number of authors cited for individual papers in high impact factor prestigious journals [5]. It is uncertain whether this same trend occurs to the same extent in minor specialty journals. Journals focusing on a specific small medical specialty – occupational medicine, were therefore selected for analysis with regard to single or multiple-authorship per peer-reviewed paper over time.

## Methods

A list of key, peer-reviewed journals was compiled from the recommended reading lists of UK academic centers involved in post-graduate training in occupational medicine, and from the American College of Occupational Medicine's recommended library [6]. Journal titles referring to 'occupational medicine' or 'work and health', were included. Journals dealing solely with environmental health, occupational hygiene, or environmental research were excluded. A further requirement was that selected journals must be listed in PubMed [7], must have a sufficient number of published articles (at least 1,500 papers); and have been allocated an impact factor by Journal Citation Reports [8]. Six occupational medicine journals were selected based on the above criteria.

Data collection and transformation was performed in three phases: 1. Collection of raw data from PubMed, 2. extraction of data required, and 3. transformation of extracted data into a suitable form for analysis. Table 1 lists the journal names, Medline abbreviations, 2006 impact factors, and the number of articles published during a full calendar year following the first issue of each journal up to the end of 2007. In cases where a journal had changed its name, all data published under the previous title were also selected and included in the analysis. This was the case for two of the six journals, namely Occupational and Environmental Medicine (previously published as the British Journal of Industrial Medicine), and Occupational Medicine (Oxford) (previously known as the Journal of the Society of Occupational Medicine).

**Table 1:** Six occupational medicine journals chosen for multiple authorship analysis

**Journal name**	**Medline abbreviation**	**Journal dates**	**2006 impact factor **[8]	**Number of articles found**
*Occupational and Environmental Medicine*	Occup Environ Med	1950–2007	2.255	4825

*Journal of Occupational and Environmental Medicine*	J Occup Environ Med	1990–2007	1.942	1593

*Scandinavian Journal of Work, Environment and Health*	Scand J Work Environ Health	1970–2007	1.735	2156

*International Archives of Occupational and Environmental Health*	Int Arch Occup Environ Health	1970–2007	1.520	2692

*American Journal of Industrial Medicine*	Am J Ind Med	1970–2007	1.433	3272

*Occupational Medicine (Oxford)*	Occup Med Lond	1970–2007	0.812	1241

### 1. Collection of raw data from PubMed

The PubMed website [7] was used to collect data for the six chosen journals. The procedure involved entering the abbreviated journal name in the "for" field, selecting the "limits" link, and selecting "Journal" in the "Limited to:" pick list. Once the "Go" button was clicked, the results were displayed on screen. These appeared in "Summary" form, but since this is not a convenient form for automated extraction of required data, the "Medline" form was used instead. This was done by clicking on the "Display" pick list and selecting "Medline". The sort order "Pub Date" was selected in a similar way, to sort articles by publication date, and the "Send to" pick list was changed to "File". Complete data for each journal were obtained using the same procedure, and saved as a text file on a local computer. This data collection was performed on Jan. 6, 2008 and was performed for all issues of the journal from the first date of publication to the end of 2007.

### 2. Extraction of required data

The six downloaded text files contained data for each journal in "Medline" form. Using a self-developed Visual Basic program, the six data files were then processed and a single data file in tabular form produced containing one line for each article in each journal with the following fields: Journal name, publication year, and number of authors.

### 3. Transformation of required data into analysis form

The third step in the process was to transform the above data file into two formats suitable for analysis. The first enabled regression analysis to be performed on the average number of authors per multiple-author article. This analysis was performed for each journal per year. The data were transformed into tabular format with the following column headings:

#### Journal name; Publication year; Total authors; Total articles; Average number of authors per multiple-author article

The second format enabled regression analysis on the percentage of articles with only one author per journal per year. This required the data to be transformed into tabular format with the following column headings:

#### Journal name; Publication year; Total single author articles; Total articles; Percentage of single author articles

The first format was created by running an "average" query to find the average authors per year. The query, which was performed separately for articles with more than one author as well as articles with one author or more, was as follows:

#### SELECT Journal, Year, Sum(Authors), Sum(ArticleID), Avg(Authors) FROM Articles GROUP BY Journal, Year

The result of the query provided the average number of authors and total articles published per year per journal.

The second format was created by running a "percent" query to find the percentage of single author articles per year. The query was performed for single author articles only, and was as follows:

#### SELECT Journal, Year, Sum(OneAuthorArticles) AS TOAA, Sum(ArticleID) AS TA, TOAA*100/TA AS [Percent One Author Articles] FROM Articles GROUP BY Journal, Year

The two resulting tables are graphically represented in the results section. This method can be used for any journal indexed in PubMed. Other details on the steps of this data collection and extraction method have been published previously [1].

Publications were classified either as "Articles" or "Letters" using the Publication Type (PT) field in the "Medline" form of the articles downloaded from PubMed. "Articles" included the following publication types: original research, review articles, case reports, and clinical trials; "Letters" included: letters to the editor and comments. For each journal, the number of "Articles" and "Letters" were further subdivided into single-author articles (1) and multiple-author articles (>1).

Further analysis was performed to compare slope and average for: a) percentage of single-author articles and average of multiple-author articles in the examined journals with each other and b) with other journals examined in a previous study (BMJ, Lancet, JAMA, New English Journal of Medicine, Nature, and Science) [5]. The slope difference would provide insight into the level of increase in the average number of multiple-author articles and the level of decrease in the percentage of single-author articles. These averages would indicate which journal had an overall higher or lower frequency of single-author articles and of multiple-author articles.

## Results

Table 2 shows the total number of articles and letters per journal classified according to grouped number of authors from the inception of the journal till the end of 2007. The average number of authors per journal per article per year was calculated for journals with more than one author and again with one author or more.

**Table 2:** Total number of articles and letters per journal classified according to grouped number of authors

Journal	Authors	Articles	Letters
*Occupational and Environmental Medicine*	**1**	**820**	**337**
	**>1**	**4005**	**185**

*Journal of Occupational and Environmental Medicine*	**1**	**107**	**131**
	**>1**	**1486**	**137**

*Scandinavian Journal of Work, Environment and Health*	**1**	**429**	**88**
	**>1**	**1727**	**54**

*International Archives of Occupational and Environmental Health*	**1**	**286**	**16**
	**>1**	**2406**	**11**

*American Journal of Industrial Medicine*	**1**	**511**	**194**
	**>1**	**2761**	**117**

*Occupational Medicine (Oxford)*	**1**	**688**	**208**
	**>1**	**1080**	**90**

**Figure 1:**
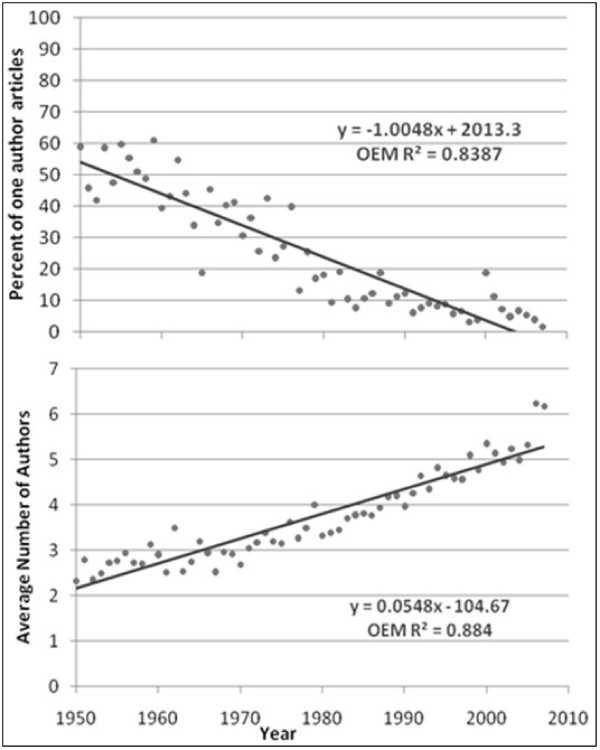
**Percentage of single-author articles and average authors per multiple-author articles for Occupational and Environmental Medicine**.

**Figure 2:**
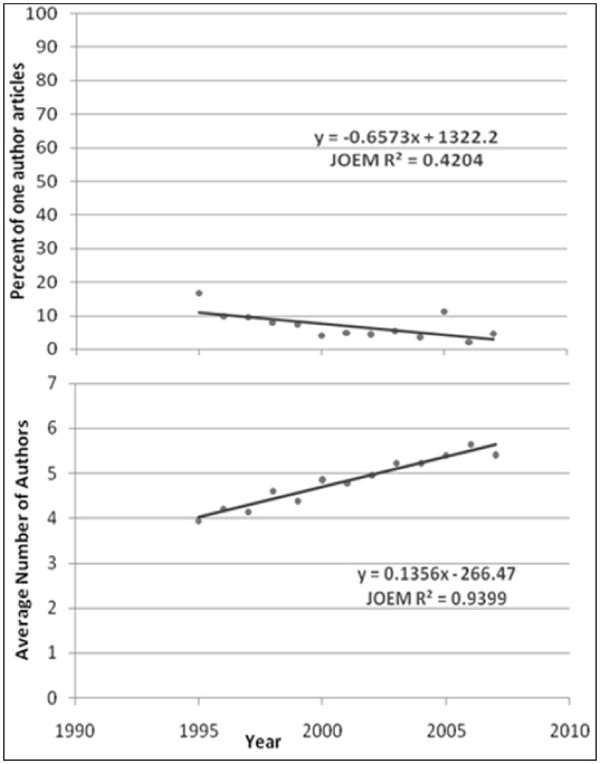
**Percentage of single-author articles and average authors per multiple-author articles for Journal of Occupational and Environmental Medicine**.

**Figure 3:**
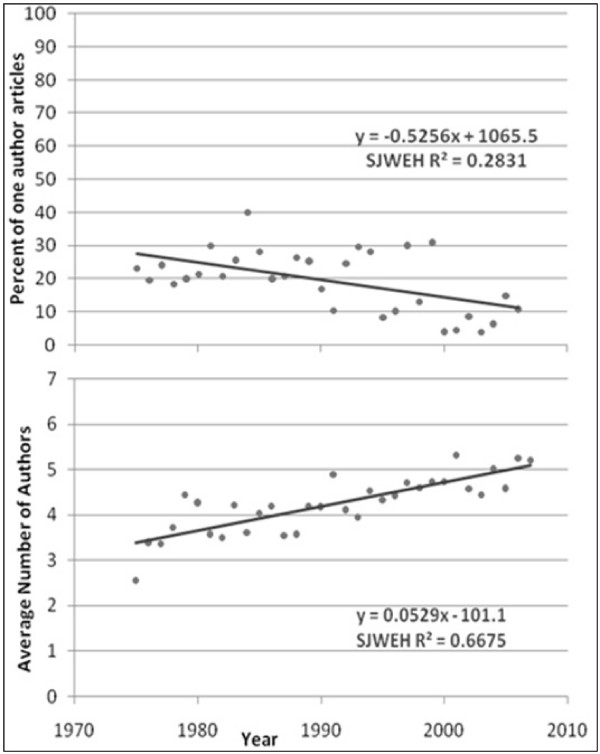
**Percentage of single-author articles and average authors per multiple-author articles for Scandinavian Journal of Work, Environment and Health**.

**Figure 4:**
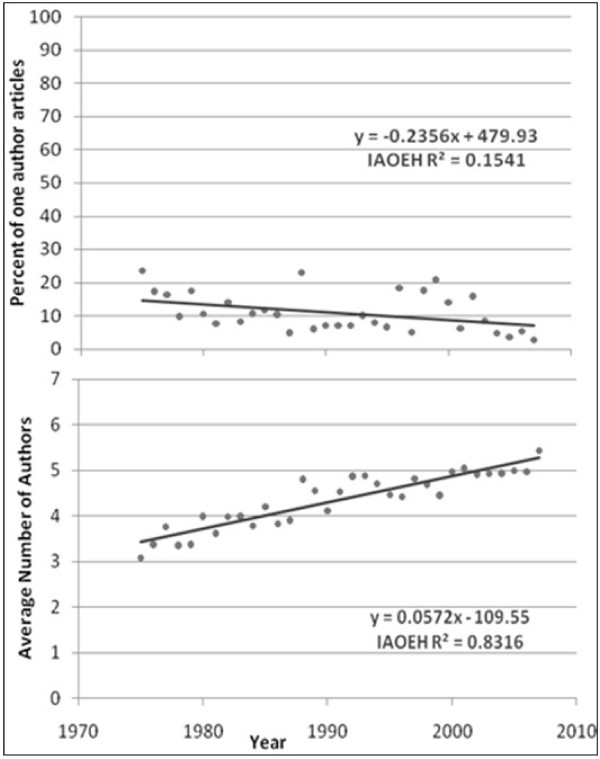
**Percentage of single-author articles and average authors per multiple-author articles for International Archives of Occupational and Environmental Health**.

**Figure 5:**
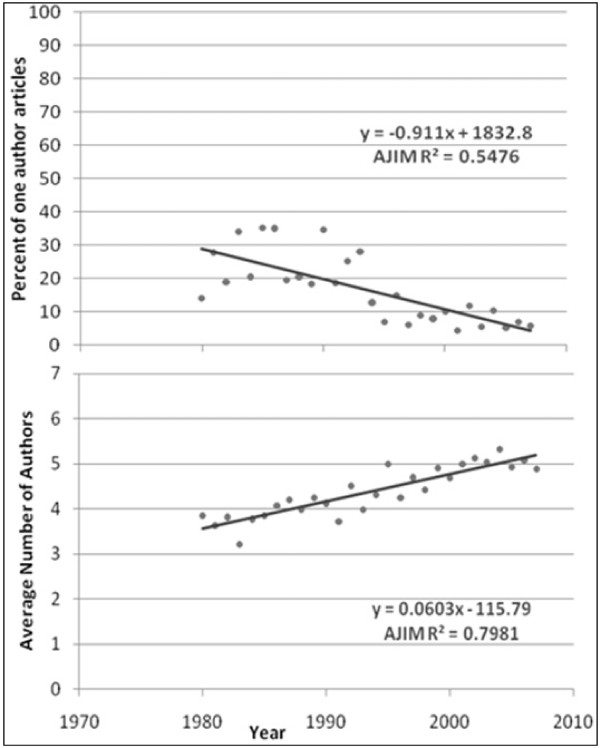
**Percentage of single-author articles and average authors per multiple-author articles for American Journal of Industrial Medicine**.

**Figure 6:**
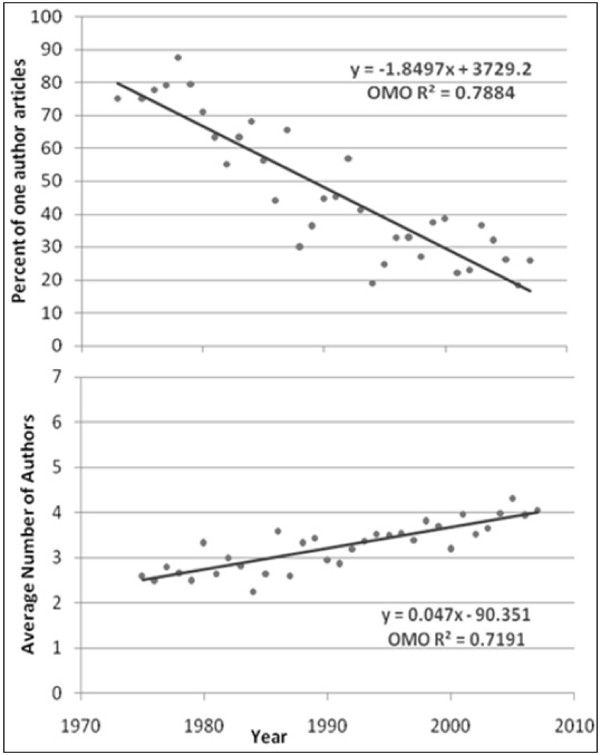
**Percentage of single-author articles and average authors per multiple-author articles for Occupational Medicine (Oxford)**.

The average number of authors for multiple-author articles is shown in Figures 1 to 6. Regression lines are shown and R^2 ^values listed. These are very high, ranging between 0.67 and 0.94, and signal a good fit to the linear model. The slope is positive (ranging from 0.047 to 0.136), indicating an increase in the average number of authors per article over the years. Of all the publications that were examined, the Journal of Occupational and Environmental Medicine had the largest slope in terms of the average number of authors per article (0.136). This is significantly higher than all other occupational medicine journals studied, where the slope ranged from 0.047 to 0.078.

The percentage of single-author articles for each journal is also given in Figures 1 to 6. Regression lines are shown, and R^2 ^values are listed. These are fairly low (ranging between 0.15 and 0.84), and signal a bad fit to the linear model for most journals. The slope, however, is clearly negative (ranging from -0.236 to -1.85), indicating a decline in single-author articles.

## Discussion

Comparison of the findings on the selected journals show that the average number of authors per article in the Journal of Occupational and Environmental Medicine is increasing at a significantly faster rate than in the other occupational medicine journals. This publication also had a significantly higher slope than all major medical journals previously studied except for JAMA and Nature. By contrast, the Journal of Occupational Medicine (Oxford), had the smallest slope (0.047), which is significantly lower than two of the other occupational journals studied, namely Occupational and Environmental Medicine and the Journal of Occupational and Environmental Medicine.

With regard to the percentage of single-author articles, we found that Occupational Medicine (Oxford) had the largest negative slope (-1.85), which is significantly higher than all other occupational journals and prestigious journals studied (-0.24 to -.91 and -0.01 to -0.42 respectively). This large difference in slope is due to the high percentage of single-author articles that appeared in OM (Oxford) during the 70's and 80's. If only papers published in the last decade are considered, the slope is calculated to be (-0.52), which shows no significant difference from other occupational medicine journals in the same period.

Occupational Medicine (Oxford), on the other hand, had the highest average of percentage of single-author articles (56.5%), significantly more than other occupational journals studied (7.0% to 19.3%) and significantly less than prestigious journals previously studied (15.3% to 34.0%). Even in the last decade, Occupational Medicine (Oxford) still had a significantly higher average of percentage of single-author articles when compared with other occupational medicine journals.

The journal with the lowest impact factor, Occupational Medicine (Oxford), is the journal with the highest percentage average of single-author articles and the lowest average of authors per article. In contrast, Occupational and Environmental Medicine, the journal with the highest impact factor, is the journal with the lowest percentage average of single-author articles and the highest average of authors per article. This is most likely due to the fact that impact factor scores are calculated according to how often articles are cited, and articles with more authors tend to be cited more frequently [9], possibly due to self-citation.

## Conclusion

The results confirm the increase in the number of authors per article in a linear fashion. They also show a decrease in the number of single-author articles but with less linear fit. These results are similar to the findings from a previous study of high impact factor journals [5]. The Journal of Occupational and Environmental Medicine has a significantly higher slope than other occupational medical journals. This suggests that this journal is increasing the number of authors per article at a more rapid pace than other journals. When we compare averages, we find that Occupational Medicine (Oxford) contains a significantly higher percentage of single-author articles than other occupational medicine journals including the high impact factor medical and health science journals previously studied. This suggests that compared with other journals, this journal is publishing a higher proportion of single-author articles. There is a direct relationship between occupational journals with higher impact factors and the higher average number of authors per article in those journals. The calculation of the impact factor should be modified so that it does not include self-citation, for as it stands, the present system may well be encouraging journals to aim for a higher impact factor rating by increasing the number of multiple-author articles they publish. There are practical and ethical issues regarding such a strategy. Procedures that may reduce the likelihood of multiple author articles include a requirement by journals for all authors to declare the extent of their contribution to the work that resulted in the papers submitted for publication. It is debatable whether the increasing trend towards multiple authorship for published papers in mainstream and smaller specialty medical journals represents a healthy development.

## Competing interests

The authors declare that they have no competing interests.

## Authors' contributions

Both authors contributed equally to the idea, background research, analysis, discussion, and conclusion sections. SS performed most of the methods sections.
